# Xylan in the Middle: Understanding Xylan Biosynthesis and Its Metabolic Dependencies Toward Improving Wood Fiber for Industrial Processing

**DOI:** 10.3389/fpls.2019.00176

**Published:** 2019-02-25

**Authors:** Martin P. Wierzbicki, Victoria Maloney, Eshchar Mizrachi, Alexander A. Myburg

**Affiliations:** Department of Biochemistry, Genetics and Microbiology, Forestry and Agricultural Biotechnology Institute (FABI), University of Pretoria, Pretoria, South Africa

**Keywords:** xylan, cellulose, lignin, wood fiber, bioproducts, biorefinery, industrial processing, metabolism

## Abstract

Lignocellulosic biomass, encompassing cellulose, lignin and hemicellulose in plant secondary cell walls (SCWs), is the most abundant source of renewable materials on earth. Currently, fast-growing woody dicots such as *Eucalyptus* and *Populus* trees are major lignocellulosic (wood fiber) feedstocks for bioproducts such as pulp, paper, cellulose, textiles, bioplastics and other biomaterials. Processing wood for these products entails separating the biomass into its three main components as efficiently as possible without compromising yield. Glucuronoxylan (xylan), the main hemicellulose present in the SCWs of hardwood trees carries chemical modifications that are associated with SCW composition and ultrastructure, and affect the recalcitrance of woody biomass to industrial processing. In this review we highlight the importance of xylan properties for industrial wood fiber processing and how gaining a greater understanding of xylan biosynthesis, specifically xylan modification, could yield novel biotechnology approaches to reduce recalcitrance or introduce novel processing traits. Altering xylan modification patterns has recently become a focus of plant SCW studies due to early findings that altered modification patterns can yield beneficial biomass processing traits. Additionally, it has been noted that plants with altered xylan composition display metabolic differences linked to changes in precursor usage. We explore the possibility of using systems biology and systems genetics approaches to gain insight into the coordination of SCW formation with other interdependent biological processes. Acetyl-CoA, s-adenosylmethionine and nucleotide sugars are precursors needed for xylan modification, however, the pathways which produce metabolic pools during different stages of fiber cell wall formation still have to be identified and their co-regulation during SCW formation elucidated. The crucial dependence on precursor metabolism provides an opportunity to alter xylan modification patterns through metabolic engineering of one or more of these interdependent pathways. The complexity of xylan biosynthesis and modification is currently a stumbling point, but it may provide new avenues for woody biomass engineering that are not possible for other biopolymers.

## Introduction

Lignocellulosic biomass from softwood and hardwood trees is most commonly used for construction, pulp and paper products and for biorefinery applications that entail separating the biomass into its individual components to produce various bioproducts (Xavier et al., 2010; Pei et al., 2016; Zhu et al., 2016). For over a century, the pulping industry has been mechanically or chemically deconstructing wood from hardwoods such as *Eucalyptus* and softwoods such as pine and spruce to produce paper and packaging products (Goswami et al., 1996; Sixta, 2006). Similar chemical processing (alkaline Kraft pulping with acidic pretreatment or acidic sulphite pulping) can be used to obtain high quality and purity cellulose for use in textiles, industrial fiber, films, food casings, plastic and various pharmaceutical related products (Klemm et al., 2005; Sixta, 2006; Sixta et al., 2013; Nasatto et al., 2015; Zhu et al., 2016). The spent chemical waste known as black (Kraft pulping) or brown (sulphite pulping) liquor can also be processed to extract valuable bioproducts such as monosaccharides, lignosulphonates and bioethanol rather than burning it to generate the heat needed for pulping liquor recovery (Hocking, 1997; Restolho et al., 2009; Xavier et al., 2010). Alternatively, after chemical or enzymatic pretreatment, the cellulosic and hemicellulosic component of lignocellulosic biomass can be subjected to saccharification and fermentation; a process which employs chemicals, enzymes and microbes to convert the polysaccharide components into ethanol for second generation biofuels and various bioproducts (Ragauskas et al., 2014).

Product value in these industries is driven by high product quality and purity, but the physical properties of the SCW biopolymers themselves impede the efficiency of deconstructing the biomass (Gübitz et al., 1998; Himmel et al., 2007; DeMartini et al., 2013; McCann and Carpita, 2015). However, several improvements have been made to woody fiber biomass processing techniques themselves which have resulted in more efficient biomass separation and higher yields (Bibi et al., 2014; Nordwald et al., 2014; Roselli et al., 2014; Chen J. et al., 2017; Shahid et al., 2017). If biomass crops which have been bred or genetically engineered for favorable processing traits were used as well, even higher yields coupled with reductions in processing costs could be achieved (Marriott et al., 2016; Zhou et al., 2017). These improvements are largely due to research that has identified genes involved in the biosynthesis and deposition of SCW biopolymers as well as the transcriptional regulation governing these processes (Persson et al., 2005; Mutwil et al., 2009; Ruprecht et al., 2011; Taylor-Teeples et al., 2015). Such research has largely been made possible by an increase in resources available for functional genomics (Oikawa et al., 2010; Gille et al., 2011a; Jensen et al., 2014), reverse genetics (Fan et al., 2015; Zhou et al., 2015; Park et al., 2017) and multi-omics approaches such as systems biology (Hillmer, 2015) analysis (Vanholme et al., 2012; Li Z. et al., 2016; Ohtani et al., 2016). The latter approach has shed valuable insight on how SCW formation is coordinated with other biological processes, what aspects of central metabolism are being drawn on and which pathways could potentially be manipulated to alter SCW polymer abundance or composition (Mizrachi et al., 2017). Systems biology approaches have been applied successfully to cellulose and lignin biosynthesis and has yielded valuable insight into their biosynthesis, regulation and metabolic dependencies. Despite these studies highlighting certain aspects of xylan biosynthesis, a xylan-centric systems biology analysis still needs to be performed to gain a holistic understanding of the process.

Xylan, the dominant hemicellulose in hardwood biomass has been identified as a major determinant of recalcitrance, yet comparably little is known about the genetic regulation and metabolic processes governing its biosynthesis, especially in woody plants (Vanholme et al., 2012; Mizrachi et al., 2013; Yen et al., 2013). The xylan polymer is composed of a repeating β1,4 xylose residue backbone, a reducing end sequence (RES) of xylose, rhamnose and galacturonic acid and is highly modified with acetyl and (methyl)glucuronic acid side groups (Scheller and Ulvskov, 2010; Pauly et al., 2013; Rennie and Scheller, 2014). Considerable progress has been made in identifying the genes involved in xylan biosynthesis (backbone elongation, RES synthesis and modification) as well as the metabolic pathways producing precursors which are used during these processes (Pauly and Scheller, 2000; Brown et al., 2007; Urbanowicz et al., 2012; Jensen et al., 2014; Marriott et al., 2016; Zhong et al., 2017). However, the biosynthetic process itself is still poorly understood as xylan knock-out mutants often have severely stunted growth (Wu et al., 2010). Physical interactions between xylan biosynthetic proteins are weak (Lund et al., 2015; Zeng et al., 2016), the proteins which interact with each other differ between species (Jiang et al., 2016; Zeng et al., 2016), and the membrane bound nature of these proteins makes *in vitro* studies difficult (Urbanowicz et al., 2014; Zhong et al., 2017). Additionally, the effects of metabolic changes on xylan biosynthesis and modification are poorly understood and have only been explored in terms of carbon supply in the form of nucleotide sugar abundance (Ishihara et al., 2015; Fan et al., 2017). The modification patterns present on the xylan backbone, which are required for tight interaction with cellulose, differ from the patterns required for an interaction with lignin (Rennie and Scheller, 2014; Busse-Wicher et al., 2016a; Giummarella and Lawoko, 2016; Grantham et al., 2017; Pereira et al., 2017; Martínez-Abad et al., 2018), which begs the question whether specific combinations of modification genes are needed to set up the patterns required for each interaction. Due to the large number of genes involved in or affecting xylan biosynthesis, discovering which of these genes are co-regulated with either cellulose or lignin biosynthesis during the different stages of xylem development could provide valuable insight into which genes are responsible for the spatial and temporal changes in xylan biosynthesis and modification (Hertzberg et al., 2001; Peralta et al., 2017).

Here we focus on xylan biosynthesis and its importance for industrial processing of lignocellulosic biomass derived from dicot wood fiber. Xylan properties play important roles in biomass recalcitrance and are therefore valuable to understand from an industrial point of view. We discuss value-added products that can be derived from lignocellulosic biomass, as well as the industrial processes pertinent to these products. We discuss xylan biotechnology from two perspectives, firstly through altering the biosynthetic process by targeting the genes directly involved, and secondly through engineering of interdependent metabolic pathways. We expand on systems biology approaches that can be used to identify pathways producing nucleotide sugars, SAM and acetyl-CoA precursors required for xylan biosynthesis and modification. Finally, we propose xylan biotechnology approaches, including altering metabolic precursor supply, which can be used to obtain novel, industrially beneficial wood fiber properties.

## Xylan and Its Role in the Secondary Cell Wall

**FIGURE 1:**
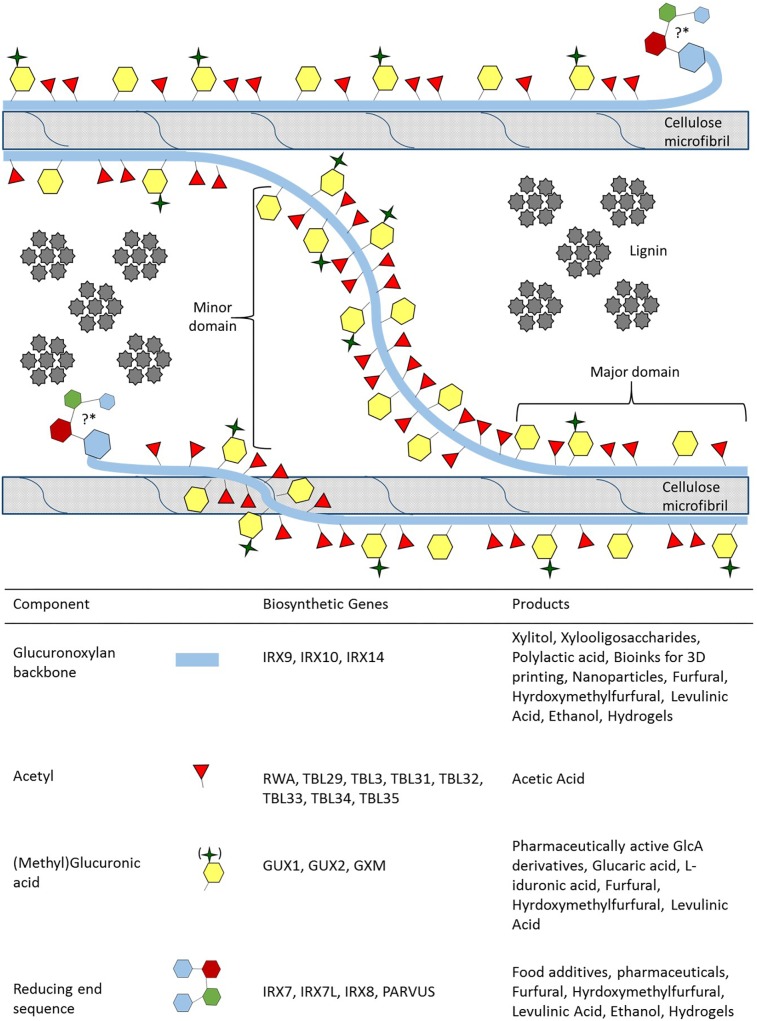
Xylan biosynthesis, its role in the secondary cell wall and xylan derived value-added products. Simplified representation of xylan, its domains and its function in the secondary cell wall, accurate modification spacing and chemical representations have been excellently review in Marriott et al. (2016) and Smith et al. (2017). Xylan can adopt two different conformations in order to interact with cellulose and lignin. The major domain of xylan is distinguished by modifications which are evenly spaced so that they face in the same direction aiding xylan in adopting a twofold helical screw conformation in order to directly interact with cellulose. The minor domain adopts the threefold helical screw conformation and acts as a linker between microfibrils, while the closely and unevenly spaced modifications play a role in establishing a hydrophobic pocket for monolignols to polymerise into lignin (Busse-Wicher et al., 2014, 2016a; Johnson et al., 2017). The position and the function of the reducing end sequence (RES) in the secondary cell wall is currently unknown, denoted by the “?^∗^”. We hypothesize the RES position and that it may have a role in attaching to arabinogalactan proteins, interacting with pectic polysaccharides and influences lignin polymerization (Tan et al., 2013; Hao et al., 2014; Biswal et al., 2015, 2018). For each component of xylan, the genes responsible for biosynthesis, and the value-added product that can be obtained are provided.

In woody dicots, the dominant hemicellulose is glucuronoxylan, however, small amounts of glucomannan and trace amount of pectins are also found in dicot SCWs (Scheller and Ulvskov, 2010). Water conducting and mechanically supportive tissues, such as the secondary xylem of woody plants, have thickened SCWs consisting of cellulose microfibrils crosslinked with hemicelluloses and fortified by a complex heterogenous matrix of lignin (Myburg et al., 2013; Plomion et al., 2001; Figure 1). The SCW is comprised of three layers: S1, S2 and S3; each of these layers has cellulose laid down at a different angle (referred to as the microfibril angle) as well as different thicknesses with the S2 layer being much thicker than S1 and S3 (Wardrop and Preston, 1947; Myburg et al., 2013). The angle of these microfibrils is determined by the orientation of the cortical microtubules as well as by hemicellulose crosslinking (Reis and Vian, 2004; Paredez et al., 2006; Watanabe et al., 2015; Schneider et al., 2017). Glucuronoxylan (xylan for short) is composed of a repeating β1,4 linked xylose backbone which is highly modified with either GlcA, which can also be methylated (MeGlcA), or an acetyl group, and a reducing end sequence (RES) composed of xylose, rhamnose and galacturonic acid (Rennie and Scheller, 2014).

(Me)GlcA modifications occur exclusively on the O-2 position of xylose residues, whereas acetyl groups can occur on both O-2 and O-3 positions with modifications being either evenly or unevenly spaced across xylose residues (Ebringerová and Heinze, 2000; Busse-Wicher et al., 2014). The spacing of the modifications is vital to the formation of two distinct domains proposed for xylan (Bromley et al., 2013; Busse-Wicher et al., 2014; Smith et al., 2017; Figure 1). The major domain has evenly spaced modifications (from every second to every tenth residue) that allows the xylan to adopt a twofold helical screw conformation similar to the glucan chains of cellulose (Simmons et al., 2016; Grantham et al., 2017; Smith et al., 2017; Figure 1). The modification present at the O-2 position sterically reduces fluctuations in xylan backbone conformation, thus promoting stable interaction with the hydrophilic face of the cellulose microfibril via hydrogen bonding (Grantham et al., 2017; Pereira et al., 2017). More densely and unevenly spaced modifications (every fifth, sixth and seventh residue are the most common) are characteristic of the minor domain which adopts a threefold helical screw conformation (Bromley et al., 2013; Busse-Wicher et al., 2014; Simmons et al., 2016; Smith et al., 2017). The minor domain can potentially interact with the hydrophobic face of cellulose, act as a linker between microfibrils, and may create a hydrophobic space between the microfibrils for monolignol polymerisation (Figure 1). While the exact mechanism of how these two domains are formed is currently unclear, the function of each domain is vital for SCW formation. An abolishment of the evenly spaced modifications in mutants of the *Eskimo1/ TRICHOME BIREFRINGENCE-LIKE (TBL) 29* gene *(esk1* in *Arabidopsis)*lead to thin SCWs, collapsed vessels and stunted growth that is characteristic of the *irregular xylem* (*irx*) phenotype (Xiong et al., 2013; Yuan et al., 2013; Grantham et al., 2017). Furthermore, altered acetylation in both domains leads to altered lignin composition (Pawar et al., 2017a,b; Yang et al., 2017). The occurrence and the type of interactions (covalent or hydrogen bonding) between xylan, glucomannan and pectin in the SCW remains to be fully resolved. Additionally, the types of linkages in lignin are thought to be influenced by polysaccharides in close proximity of the monolignols during polymerisation (Lairez et al., 2005; Li et al., 2015). Therefore, both the interaction of polysaccharides and the modification patterns on the xylan backbone may influence lignin composition. It is therefore likely that, in addition to xylan’s role in stabilizing and orientating cellulose microfibrils, but it also affects lignin content, solubility and composition. Consequently, xylan traits are often strongly correlated with processability traits (Hao et al., 2014; Johnson et al., 2017; Lyczakowski et al., 2017). A greater understanding of xylan biosynthesis and modification would therefore be useful to engineer woody biomass for improved processing traits.

## Biorefinery Associated Industrial Processes

### Industrial Processes

Due to the abundance and renewable nature of wood, it has always been a sought after natural commodity. Historical evidence shows that wood was used for structural timber as long as 2.6 million years ago (Klein and Grabner, 2015). The use of wood pulp as a paper source is a much more recent innovation with commercial-scale wood pulp and paper production starting with the invention of large, industrial mechanical pulping plants in the 1840s (Hunter and De la Mare, 1978). Soon after the wide-scale adoption of mechanical pulping, chemical pulping became common with sulphite pulping being the dominant form until the 1940’s when sulfate (kraft) pulping with the addition of pre-boilers became prevalent and today remains the preferred pulping process for paper production. During the pulping process, lignin is removed from the biomass leaving behind cellulose and xylan. The pulp is most often used for paper production and, due to its renewable nature, it is also increasingly being used for the manufacturing of paper bags and boxes for packaging as an alternative to non-renewable plastics. The lignin which is removed during pulping is, due to its high calorific value, typically burned to produce the heat required for the pulping process. Recently, rather than burning, lignin is isolated for the production of various value added products. Industries which process wood fiber derived biomass have increasingly begun adopting biorefinery approaches toward converting all of the wood biomass into a wide variety of valuable renewable bioproducts (products listed in Table 1). Processing plants aim to either obtain the three individual components of the biomass for biopolymer or biorefinery applications, or for simple sugars for fermentation into biofuels (Klemm et al., 2005; Restolho et al., 2009; Ragauskas et al., 2014; Nasatto et al., 2015). However, due to the recalcitrance of lignocellulosic biomass, industrial deconstruction has had to deal with several costly hurdles to increase quality and quantity of the desired constituents (Sixta, 2006; Himmel et al., 2007).

**Table 1:** Xylan as a source of recalcitrance to woody biomass processing and improvements being made to reduce recalcitrance.

	Saccharification and Fermentation (S&F)	Dissolving pulp production (DPP)
Main product(s)	• Ethanol [1,2,3].	• Pure cellulose: nanocellulose viscose (textiles), rayon (tire strings), cellulose acetate films, methylcellulose, nanopaper, surgical stitches [2,4,5,6].
Value added Products from “waste”	• Bioplastics, fermentable lignin, pharmaceuticals, flavourants [2].	• Xylitol, lignosulphonates, bioinks, nanoparticles, pharmaceuticals, bioplastics [5,7,8,9].
Xylans Impact on the industrial process	• Blockage of glucanase access to cellulose as a result of xylan major domain’s tight association with cellulose reduces saccharification efficiency [10]. • Pre-treatment causes xylan present on the hydrophilic face of cellulose to slide to the hydrophobic face through the action of GlucA .groups, which facilitates additional chemical treatment to remove [12]. • Lignin carbohydrate complexes formed during pre-treatment block xylanases [13, 14, 15, 16] • Pre-treatment causes undesirable and toxic breakdown products which inhibit fermentation into ethanol [17,18]. • Acetyl groups blocking CWDE [19, 20, 21]. • Released acetyl groups alter pH of fermentation fluid thereby inhibiting fermentation [17,18]. • GlucA methylation affects xylose release [27]. • Yeast does not ferment xylose efficiently [17,18].	• Xylan major domain’s tight association with cellulose reduces separation and purity of biopolymers [11,12]. • Pre-treatment causes xylan present on the hydrophilic face of cellulose to slide to the hydrophobic face through the action of GlucA groups, which facilitates additional chemical treatment to remove [12]. • Lignin carbohydrate complexes formed during pre-treatment block efficient chemical removal [13,14,15,16] • Pre-treatment causes undesirable and toxic breakdown products which decrease the purity of the separated biopolymers [4] • Strong acid treatment decreases strength of cellulose fibers due to cellulose autohydrolysis [4,22,23]. • Released acetyl groups alter pH of alkaline pulping liquors [24,25,26]. • Calcium bridges and crystal formation around adjacent GlucA groups lead to the use of strong acids for xvlan removal [12]. • Stacking of multiple xylan chain increase stability xylans association with cellulose [12].
Improvements to techniques	• Ionic liquid and microwave assisted heating used during pretreatment increase biomass separation while decreasing toxin and inhibitor production [28,29,30,31]. • Multifunctional enzymes [32.33,34]. • Genetically engineered yeasts [38,39,40,41].	• Ionic liquids allow for improved biopolymer separation [28,29,30,31]. • Improved cellulose fibrillation with chemical treatment with 2,2,6,6- tetramethylpiperidine-l-oxyl (TEMPO) [35,36,37]. • WET spinning small cellulose fragments into large [42,43].
Plant biotechnology approaches	Down-regulation of recalcitrance associated genes [44,45,46,47]. Upregulation of recalcitrance reducing genes [48,49]. Knock-out mutagenesis of recalcitrance associated genes [27,50,51]. Vessel complementation of knock-out mutants [52]. Ectopic xylan modifications [53,54,55]. Metabolic engineering [56,57,58,59,60,61] CRISPR:CAS9,dCAS9, or activator and DNA methyltransferase fusion dCAS9 [62,63,64]. Endogenous expression of processing enzymes which can also become active only under specific conditions | 65,66.67,68,69]. Promoter feedback loops [70]. Gene stacking [71].


*References pertaining to numbered items can be found in Supplementary File S2*.

**FIGURE 2:**
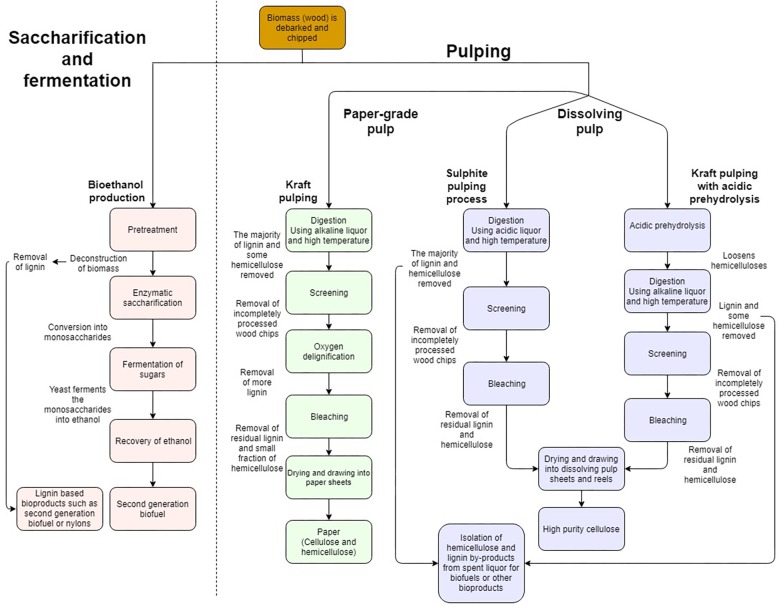
Simplified comparison of industrial processes such as pulping or saccharification and fermentation, which produce renewable bioproducts from lignocellulosic biomass. Comparison of the processing steps where woody biomass is converted into bioproducts. Pulping results in either paper-grade pulp which comprises both cellulose and hemicellulose with lignin removed, or dissolving pulp where both lignin and hemicelluloses are removed to yield only high purity cellulose. Due to the simplified nature of the illustration, several processing steps may be represented as a single process.

The industrial processing of wood into value added products involves two main approaches summarized in Figure 2. The process of saccharification and fermentation (S&F) refers to the separation of SCW polymers in lignocellulosic biomass through pretreatment, subsequent digestion of the polymers by cell wall degrading enzymes (CWDE) to liberate monomeric cell wall sugars and, ultimately, fermentation of the sugars by microbes to produce bioethanol (Doran-Peterson et al., 2009; Ragauskas et al., 2014; Cheng et al., 2017; Figure 2). In contrast, dissolving pulp processing (DPP) is purely chemical in nature where either highly acidic (sulphite pulping) or basic liquors (Kraft pulping) are used to separate the xylan and lignin from the cellulose at high temperatures yielding only cellulose of high quality and purity (Figure 2). The main aim of S&F industries is to produce the highest possible yield of ethanol at the lowest possible price, whereas chemical cellulose of large degree of polymerization (DP) with high purity and quality is required for cellulose derived products from DPP. These products include multiple new applications for cellulose, such as aerogels, resin impregnated fibers as well as macrofibers which display optical, magnetic and electrical properties. These products are derived from nanofibrillated cellulose (NFC), short cellulose chains which are chemically treated to gain new properties (Walther et al., 2011; Zhu et al., 2016). To accomplish these aims, xylan, lignin, contaminants and inhibitors need to be separated into distinct processing streams for their eventual use as biorefinery feedstocks (Xavier et al., 2010; Vardon et al., 2016). During polymer separation, lignin can also be liberated for applications other than fuels (Table 1). Unfortunately, the separation in the different product components for both S&F and DPP has faced many challenges on the path to commercial viability.

### Xylan’s Impact on Industrial Processing

Native xylan properties play a significant role in recalcitrance. Efficient separation of xylan from cellulose is a major obstacle for both S&F and DPP as the association is stabilized on multiple levels namely, the backbone itself (Pereira et al., 2017), the spacing of modifications on the backbone (Simmons et al., 2016; Grantham et al., 2017; Pereira et al., 2017) as well as the stabilizing effect of adjacent GlcA modifications that associate via chelating Ca^2+^ ions *in muro* (Pereira et al., 2017; Table 1). Interactions between xylan and lignin are less understood, but the quantity of acetyl modifications as well as methylation of GlcA influences lignin composition and solubility (Urbanowicz et al., 2012; Giummarella and Lawoko, 2016; Johnson et al., 2017; Pawar et al., 2017a,b), whereas lignin composition affects release of xylan side groups (Van Acker et al., 2017; Table 1). Modifications on the xylan backbone block access of cell wall degrading enzymes to the biopolymer substrate, which affects the polysaccharide monomerisation in S&F and reduces the efficiency of residual xylan removal by xylanases used by DPP industries as an approach to upgrade chemical cellulose quality (Helle et al., 2003; Qing and Wyman, 2011; Jönsson et al., 2013; Chong et al., 2015; Table 1). Deconstruction and monomerisation of xylan also has a role in recalcitrance as the yeast used (in S&F) to ferment sugars into ethanol do not ferment xylose efficiently and the released acetyl groups alter pH of the fermentation medium in S&F (Rastogi and Shrivastava, 2017; Yusuf and Gaur, 2017) and pulping liquor in alkaline based DPP (Kobayashi et al., 1960; Lacerda et al., 2013; Dong et al., 2016; Table 1). The abovementioned recalcitrance mechanisms require S&F and DPP industries to perform pretreatment, perform additional chemical treatments while constantly replenishing CWDE and yeast to maintain monomerisation and fermentation efficiency.

Pretreatment of biomass is required for biopolymer separation, however, the effect of industrial processing on xylan contributes to a separate set of recalcitrance associated factors. During the heating phase of pretreatment, xylan “slides” from the hydrophilic to the hydrophobic face of cellulose as a result of the GlucA modifications rather than being released (Busse-Wicher et al., 2016b; Pereira et al., 2017), resulting in incomplete separation, thus requiring additional treatment to remove the residual xylan. Adding strong sulphuric acid to dissolving pulps is a strategy to remove residual xylan, but this causes cellulose autohydrolysis; leading to a reduction in cellulose quantity and DP as well as a subsequent reduction in the tensile strength of the resulting product (Sixta, 2006; Arola et al., 2013; Stepan et al., 2016). An alternative measure to remove residual xylan is to treat the extracted cellulose with xylanases, however, xylanase activity is inhibited by the presence of lignin-carbohydrate complexes (LCC), physical (covalent) linkages between xylan and lignin, created either through the harsh chemical treatments or occurring natively in biomass (Gierer and Wännström, 1986; Iversen and Wännström, 1986; Gübitz et al., 1998; Giummarella and Lawoko, 2016; Nishimura et al., 2018). Furthermore, positive metal ions in ionic buffers used during pretreatment react with the calcium which bridge two GlucAs to form a crystal which further limits biopolymer separation (Pereira et al., 2017). Heat and pretreatment create by-products which are fermentation inhibitors and contribute to loss in total yield for biorefinery-centered DPP (Sixta, 2006; Rastogi and Shrivastava, 2017; Yusuf and Gaur, 2017 which facilitates the need to improve both the industrial techniques as well as plant biotechnology targeting xylan biosynthesis.

### Improvements to the Industrial Process Methodology

One main improvement that has benefited both S&F and DPP is the use of several ionic liquid (IL) solvents that function to improve dissolution and the separation properties of the lignocellulosic biomass (Roselli et al., 2014; Chen J. et al., 2017). Pulps produced using IL solvents display improved digestion by xylanases which results in higher cellulose quality by reducing residual xylan contamination (Roselli et al., 2014), while enzyme cocktails repertoires for S&F have expanded to contain CWDE which are thermally stable, inhibitor insensitive and multifunctional (Bibi et al., 2014; Nordwald et al., 2014; Shahid et al., 2017); (Table 1). Furthermore, S&F has greatly benefitted from genetically engineered yeasts, which ferment pentoses, break down phenolic inhibitors by secreting laccases, as well as being tolerant to changing pH and growth inhibitors (Larsson et al., 2001; Jin and Jeffries, 2003; Jin et al., 2003; Wei et al., 2013; Table 1). Moreover, the issue with the creation of shorter DP cellulose can potentially be circumvented by using the reducing agent 2,2,6,6-tetramethylpiperidine-1-oxyl (TEMPO) to create nanofibrillated cellulose (NFC), small fragments of cellulose that maintain its mechanical properties (Saito and Isogai, 2004; Saito et al., 2007; Isogai et al., 2011; Table 1). These NFC fragments can be chemically combined to reconstitute large macrofibers via wet-spinning using a syringe and coagulation bath, which is beneficial as mechanical strength of the chemical cellulose is retained and novel properties can be achieved by impregnation with various chemicals and resins (Iwamoto et al., 2011; Walther et al., 2011; Table 1). Improvements to the techniques have largely benefitted production, however, additional yield improvements and price reduction may be attained by making use of plants which produce biomass with reduced recalcitrance and beneficial processing traits.

### Biotechnology Approaches to Improve Industrial Processing

Plant biotechnology approaches aimed at altering endogenous xylan biosynthesis genes (i.e., cisgenic approaches) have shown that targeting xylan properties can result in favorable phenotypes related to plant growth, SCW composition and industrial processing efficiency. Altering expression of genes coding for proteins related to xylan biosynthesis has proven to be an effective strategy. Favorable phenotypes have been observed in *Populus* as a result of downregulation targeting backbone and RES genes (Biswal et al., 2015; Ratke et al., 2018), as well as overexpression and downregulation of xylan modification genes (Song et al., 2015; Pawar et al., 2017b; Yang et al., 2017; Table 1). Studies in *Arabidopsis* based on knock-out mutagenesis has yielded positive industrial processing traits when targeting genes associated with GlcA addition as well as GlcA methylation (Mortimer et al., 2010; Urbanowicz et al., 2012; Lyczakowski et al., 2017), whereas *irx* phenotypes were obtained in knock-outs of backbone, RES and some acetylation related genes (Brown et al., 2007; Peña et al., 2007; Lee et al., 2012c; Manabe et al., 2013; Xiong et al., 2013; Yuan et al., 2013; Schultink et al., 2015; Table 1). Growth of *Arabidopsis* xylan related *irx* mutant plants has been restored, while retaining positive processing traits by using a VND7 promoter that complements the expression of the knocked-out gene in vessel cells (Petersen et al., 2012; Table 1). Efficacy of this approach in woody plants remains to be seen. Inducing increased SCW formation in general through positive feedback loops (Yang et al., 2013) as well as altering metabolic flux to SCW wall biopolymer biosynthesis (Tauberger et al., 1999; Fan et al., 2017) has yielded SCW properties that are beneficial to industrial processing, and can be achieved using a wild type or mutant background (Yang et al., 2013; Table 1). The progress currently made in cisgenics now opens the door for the use of novel techniques such as CRISPR, where different CAS9 enzymes can be used to achieve various effects, such as knock-out mutations using standard CAS9 (Fan et al., 2015; Zhou et al., 2015; Park et al., 2017) or altering expression using either dCAS9 or modified dCAS9 fusion proteins (Jinek et al., 2012; Gilbert et al., 2013; Qi et al., 2013; Vojta et al., 2016; Xiong et al., 2017; Table 1).

Progress has also been made in plant engineering efforts relying on heterologous expression of xylan biosynthesis and modification genes. Such transgenic approaches have not only produced favorable phenotypes for industrial processing, but have resulted in plants with novel SCW properties as well as additional traits such as increased stress tolerance. Self-processing plants with increased industrial processing efficiency and biotic defense responses are generated by *in planta* expression of xylan targeting CWDE from wood rotting fungi that can be active under normal plant growth (Gandla et al., 2014; Pawar et al., 2017b), or only become active upon application of external stimulus (Shen et al., 2012; Mir et al., 2017; Xiao et al., 2018; Table 1). Various studies have shown that xylan modifications differ between plant species and lineages (Allerdings et al., 2006; Rennie and Scheller, 2014; Peña et al., 2016; Smith et al., 2017; de Souza et al., 2018) and modification genes from one lineage can add modifications to the xylan backbone of another (Anders et al., 2012; Xiong et al., 2015; Zhong et al., 2018; Table 1). This field has not been thoroughly explored, but may be a novel manner in which to alter SCW traits. The ability to alter SCW composition and several SCW traits simultaneously has emerged due to recent breakthrough in gene stacking, where various genetic background can be used to not only achieved designer biomass (Gondolf et al., 2014; Eudes et al., 2015, 2016; Smith et al., 2017; Aznar et al., 2018; Table 1).

The above genetic engineering efforts have primarily been tested in *Arabidopsis*, but several studies have shown that SCW engineering approaches have scaled successfully to *Populus* (Supplementary File S3). The availability of reference genomes and the possibility of genetic transformation means that similar approaches can be applied and validated on woody biomass crops such as *Populus* (Song et al., 2006; Tuskan et al., 2006; Maheshwari and Kovalchuk, 2016) and *Eucalyptus* (Mullins et al., 1997; Myburg et al., 2013, 2014; Klocko et al., 2016). Engineering of SCW traits has been focused on reducing recalcitrance to optimize ethanol yield for S&F industries. Biomass from these modified plants has rarely been used for pulping or DPP applications (Zhou et al., 2017). However, reductions in residual xylan in dissolving pulps may decrease processing costs, thereby increasing the feasibility of replacing many petroleum derived products with alternatives derived from woody biomass.

## Novel Biotechnology Strategies

### Strategies That Target Xylan Biosynthesis Genes

Unlike cellulose, xylan is synthesized in the Golgi with other hemicelluloses (xyloglucan and glucomannan) and pectins (RGI and RGII), with biosynthesis thought to occur in the medial Golgi network (Scheller and Ulvskov, 2010; Kim and Brandizzi, 2016; Meents et al., 2018). Xylan is synthesized by membrane bound proteins which are responsible for forming the backbone, RES and backbone modifications, and rely on nucleotide sugar, acetyl-CoA as well as S-adenosylmethionine (SAM) precursors that are transported into the Golgi from the cytosol (Pauly and Scheller, 2000; Manabe et al., 2013; Rennie and Scheller, 2014; Ebert et al., 2015). The modifications on the dicot xylan backbone can occur as six possible structural configurations: 2-O monoacetylation, 3-O monoacetylation, 2,3-O diacetylation, α1,2-linked D-GlcA, α1,2-linked 4-O-MeGlcA and 3-O acetylation with α1,2-(Me)GlcA (Ebringerová and Heinze, 2000; Yuan et al., 2013). It is currently accepted that, when biosynthesis is completed, the xylan is packaged in to vesicles in the *trans*-Golgi and transported to the cell wall. Whether any additional modification to xylan occurs while being transported is unknown. Upon arrival at the cell wall, xylan coats and crosslinks the cellulose microfibrils (Kabel et al., 2007; Busse-Wicher et al., 2014; Simmons et al., 2016). Modification of xylan structure occurs through the action of cell wall bound xylanases and transglycosylases in response to stress (abiotic, biotic and tension) and this can happen throughout SCW formation (Minic et al., 2004; Derba-Maceluch et al., 2015). The modification pattern on xylan has also been shown to differ spatially and temporally throughout stem development (Peralta et al., 2017), but it is not clear whether any of these patterns are altered after deposition into the cell wall. Xylan biosynthesis has been thoroughly reviewed in the past (Rennie and Scheller, 2014; Marriott et al., 2016; Smith et al., 2017), and we therefore only focus on some potential implications of recent studies and how these could lead to novel biotechnology applications. We aim to stimulate hypothesis testing in the xylan research community with some of the ideas we put forward in the subsequent sections.

#### Xylan Backbone Biosynthesis

Elongation of the xylan backbone is performed by the xylan synthase complex (XSC). XSC related genes have been implicated as recalcitrance factors and recent studies related to the expression of XSC genes point to interesting avenues for further investigation. The XSC is comprised of IRX9, IRX10, and IRX14, these proteins are likely interacting with one another physically, have non-redundant roles and the genes encoding the XSC proteins are preferentially expressed in SCW forming tissues (Wu et al., 2010; Zeng et al., 2010, 2016; Ren et al., 2014; Jiang et al., 2016; Ratke et al., 2018; Figure 1). Additionally, three homologs of the XSC proteins (IRX9L, IRX10L, and IRX14L) have been identified and shown to be involved in the synthesis of PCW xylan indicating two different sets of XSCs for PCW and SCW formation (Mortimer et al., 2015). It has, however, been noted that a SCW XSC mutant can be partially complemented by its PCW XSC homolog when expression is driven by the promoter of the SCW homolog and vice versa (Wu et al., 2010; Mortimer et al., 2015). As expected, the expression of the PCW XSC genes differs from the genes associated with the SCW XSC, but SCW XSC genes also have slightly different expression patterns from each other during SCW formation (Li Z. et al., 2016; Sundell et al., 2017). Expression of these genes is essential for normal plant development, as knocking out a XSC gene leads to dwarfing and *irx* phenotypes (Brown et al., 2007; Peña et al., 2007; Lee et al., 2012c), but more interestingly, reducing the expression of XSC genes beneficially alters plant growth as well as xylem and SCW properties (Ratke et al., 2018). Therefore, altering XSC expression, rather than completely knocking out the XSC genes, could be a productive biotechnology approach.

The expression of XSC genes has biotechnology applications by using promoters from SCW XSC genes to express genes of interest or RNA interference (RNAi) fragments specifically in SCW forming tissues (Ratke et al., 2015, 2018), but altering expression of XSC genes may also be a promising biotechnology route to pursue. RNAi mediated downregulation of IRX9 and IRX14 homologs in *Populus* using an IRX9 promoter lead to an upregulation in cell cycle genes that resulted in taller plants with an increased stem diameter and volume (Ratke et al., 2018). SCW formation was downregulated in the transgenic plants, resulting in xylem cells which had thinner and less recalcitrant SCWs, but no significant reduction in xylose content, perhaps indicating that altering XSC related expression may be a manner in which SCW traits can be changed (Ratke et al., 2018). It remains to be seen whether the abovementioned phenotype would be replicated if a promoter was used from either another XSC related gene or a gene related to another SCW process such as cellulose or lignin biosynthesis. It is also unclear whether the abovementioned phenotype would differ if IRX10 was downregulated. Despite being a contributor to recalcitrance, xylan is a valuable bioproduct of cellulose pulping that can be used for various applications such as hydrogels, nanoparticles and 3D printing bioinks (Gericke et al., 2018; Li and Su, 2018; Xu et al., 2018). It also has dietary value (Huang et al., 2018) as well as being a source of value added products such as acetone, xylitol, xylooligosaccharides, furfural, hydroxymethylfurfural, levulinic acid, polylactic acid and ethanol (Parajó et al., 1997; Zhao et al., 2007; Binder et al., 2010; Deutschmann and Dekker, 2012; Gürbüz et al., 2012; Li et al., 2013; Zhao et al., 2013; Lama et al., 2014; Figure 1). The overexpression of endogenous XSC related genes to increase xylose content has not been attempted previously and there is a need to address the possibility of a metabolic penalty. Recently it has been shown that overexpression of a single PCW associated CesA6-like gene can increase cellulose content (Hu et al., 2017). The type (PCW vs. SCW) of XSC gene that is overexpressed might therefore be a factor to consider when trying to increase xylose content. Additionally, there is a need to investigate whether overexpression of a single XSC gene would be sufficient to increase xylose content, whether co-suppression would occur and whether UDP-xylose flux have to be increased toward xylan biosynthesis?

XSC composition (XSC stoichiometry and XSC interaction with other proteins) is a recent addition to xylan biosynthesis research. Similar to the cellulose synthase (CesA) complexes, XSC stoichiometry differs between plant species (Jiang et al., 2016; Li S. et al., 2016; Zeng et al., 2016; Zhang et al., 2018). Additionally, CesA stoichiometry also differs between tissues (Zhang et al., 2018), xylosyltransferase (XylT) activity is influenced by XSC composition (Zeng et al., 2016), therefore it would be tempting to propose that tissue and species-specific differences in XylT activity (Song et al., 2015; Jiang et al., 2016) could be due to differences in XSC composition. Overexpression of IRX9 and IRX14 (GT43) homologs from cotton in *Arabidopsis* increased xylose yield, most probably due to upregulation of other xylose biosynthesis related genes (Li et al., 2014). Replacing endogenous *Arabidopsis* XSC genes with homologs from rice alters XylT activity and chain length (Chiniquy et al., 2013). XSC composition may also influence xylan modification. In wheat it has been shown that XSC interacting proteins such as UDP-arabinopyranose (Ara*p*) mutase (UAM), which reversibly converts UDP-Ara*p* into UDP-arabinofuranose (Ara*f*), produce the substrate required by xylanarabinotransferases (XAT) to add arabinose substitutions to the xylan backbone (Konishi et al., 2007; Anders et al., 2012). Additionally, two proteins annotated as vernalization-related gene 2 (VER2) and germin-like protein (GLP; Zeng et al., 2010; Jiang et al., 2016) also interact with the XSC, and are thought to be associated with the hormones jasmonic acid and auxin, respectively. The XSC physically interacting with these proteins either aids in targeting the XSC correctly to the *trans*-Golgi (GLP) or to unknown compartments (VER2), which suggests (at least in wheat) that hormone interaction with the XSC regulates SCW xylan biosynthesis (Jiang et al., 2016). Together, these results suggest that XSC composition affects xylan content, modification and transport and it would therefore be desirable to better understand the biotechnology potential of altering XSC related genes.

The results from the preliminary findings on XSC composition point to a number of novel xylan biotechnology approaches. Heterologous expression of XSC genes from other plant species seems to affect xylan properties. Additionally, introducing monocot XSC genes, UAM and XAT into a dicot system could facilitate increased incorporation of ectopic O-2 and/or O-3 arabinose modifications compared to what was previously possible (Anders et al., 2012; Xiong et al., 2013). By putting VER2 expression under the control of different SCW promoters (Tokunaga et al., 2009; Smith et al., 2013; Ratke et al., 2015), or even an inducible promoter (Gatz, 1996; Caddick et al., 1998), one could potentially engineer xylan biosynthesis by altering the transport of the XSC (Jiang et al., 2016). XSC related biotechnology approaches could allow us to have greater control over xylan biosynthesis in general from timing of biosynthesis to altering xylan properties and modifications.

#### Reducing End Sequence

The RES is a tetrasaccharide comprising of β-Xyl-(1,3)-α-Rha-(1,2)-α-GalA-(1,4)-Xyl, the function of which is currently unknown and the main discussion being whether the RES acts as a primer or terminator. We want to discuss how the RES effects xylan biosynthesis (York and O’Neill, 2008; Smith et al., 2017). The RES has been proposed to act as a primer since *in vitro* expressed IRX10 was observed to elongate the xylan backbone from the reducing end (Urbanowicz et al., 2014; Smith et al., 2017). This is rather interesting since mutation in the RES related genes, IRX7, IRX7L, IRX8 and PARVUS (Figure 1), lead to variable chain lengths and XylT activity (Brown et al., 2005, 2007; Peña et al., 2007; Lee et al., 2007, 2009; Wu et al., 2010). Plants with mutations in RES genes tend to be severely dwarfed (Rennie and Scheller, 2014), thus complicating the study of RES related genes, but a greater understanding of this process could yield biotechnology tools which could be used to alter xylan content.

Besides the dwarfing effect of RES mutants, more moderate changes in expression patterns of RES genes also affect plant development. It was observed that overexpression of IRX8 in *Populus* resulted in decreased growth and higher recalcitrance, whereas downregulation had the opposite effect (Biswal et al., 2015, 2018). It remains to be seen whether overexpression and downregulation of other RES genes result in these two opposite phenotypes. If the RES does indeed act as a primer for xylan biosynthesis (Smith et al., 2017), overexpression of the RES genes could also be used to the increase total xylose content (Biswal et al., 2018), by potentially promoting higher rates of xylan biosynthesis initiation.

The biosynthesis of xylan and the heparan sulfate (HS) proteoglycan have often drawn parallels (Smith et al., 2017), as both these polysaccharides are biosynthesised in the Golgi and possess an RES (Kreuger and Kjellén, 2012; Rennie and Scheller, 2014). The RES in HS attaches to a protein required for transport to the cell wall (Kreuger and Kjellén, 2012), so it would be tempting to suggest that perhaps xylan is also attached to a protein required for transport? Some support for this hypothesis exists as arabinogalactan proteins (AGPs) called ARABINOXYLAN PECTIN ARABINOGALACTAN PROTEIN1 (APAP1) have been found to be attached to PCW xylans (Tan et al., 2013). If the attachment of xylan to an AGP is required for transport, a lack of transport to the cell wall could be the reason for fewer xylan chains being detected in the SCW in RES mutants (Peña et al., 2007; Persson et al., 2007). In the PCW, xylan aids in the interaction of RGI with cellulose (Ralet et al., 2016), the same biopolymers which are affected in RES mutants (Zhong et al., 2005; Brown et al., 2007; Lee et al., 2007; Persson et al., 2007; Figure 1). So does the RES have a role in SCW biopolymer interaction as well? Based on the glycome profiling results obtained from the biomass of plants where IRX8 was either overexpressed or downregulated (Biswal et al., 2015, 2018), it has been suggested that that the RES associates with a homogalacturonan molecule and which may have a direct or indirect effect on guaiacyl lignin polymerization (Persson et al., 2007; Hao et al., 2014; Biswal et al., 2015, 2018; Figure 1). Taking the potential interaction of xylan with other molecules into account could be a manner in which to alter multiple traits simultaneously.

If xylan, like HS (Kreuger and Kjellén, 2012), relies on one or more proteins for transport to the cell wall, the expression of the gene(s) corresponding to the transport proteins could be put under the control of various other SCW promoters to specify the stages of SCW development at which xylan should be transported to the cell wall. Tan et al. (2013) provided the most comprehensive illustration of PCW xylan attached to an AGP. The study also illustrated that xylan was attached to the pectin molecules homogalacturonan and rhamnogalacturonan I. Keeping this structure in mind, it perhaps makes sense that galacturonic acid content was altered in response to IRX8 overexpression and downregulation (Biswal et al., 2015, 2018). Similarly, *irx7* mutants may display compromised seed coat mucilage anchoring (Hu et al., 2016) and reduced xylan content at the SCW (Brown et al., 2007) due to lack of attachments to an AGP. Effective transport of xylan may be hindered in both tissues due to a lack of an RES, but before this hypothesis can be tested, we need to determine whether SCW xylan relies on an AGP for transport the cell wall. Fasciclin-like arabinogalactan (FLA) are AGPs expressed during SCW biosynthesis and FLA mutants display differences in xylose content as well as microfibril angle (MacMillan et al., 2010, 2015). A change in microfibril angle has been noted in a few xylan mutants (Derba-Maceluch et al., 2015; Ratke et al., 2018), xylan has previously been suggested to play a role in microfibril angle of cellulose and has been suggested as a reason to why SCW patterning is maintained even when microtubule formation is disrupted (Reis and Vian, 2004; MacMillan et al., 2010; Schneider et al., 2017). Therefore, it could be possible to engineer microfibril angles required by specific industries or biorefinery applications by altering FLA expression patterns. Pectin and xylan can be converted into various value added products through biorefining (Deutschmann and Dekker, 2012; Gürbüz et al., 2012; Xiao and Anderson, 2013; Gericke et al., 2018; Figure 1), overexpressing IRX8 lead to increase in both of these biopolymers (Biswal et al., 2018). However, this plant also displayed increased biomass recalcitrance and decreased growth, if these undesirable traits could be alleviated, the IRX8 overexpressor may have biorefinery applications. The evidence we have provided suggests that there targeting RES synthesis is a viable biotechnology strategy.

#### Xylan Acetylation

**FIGURE 3:**
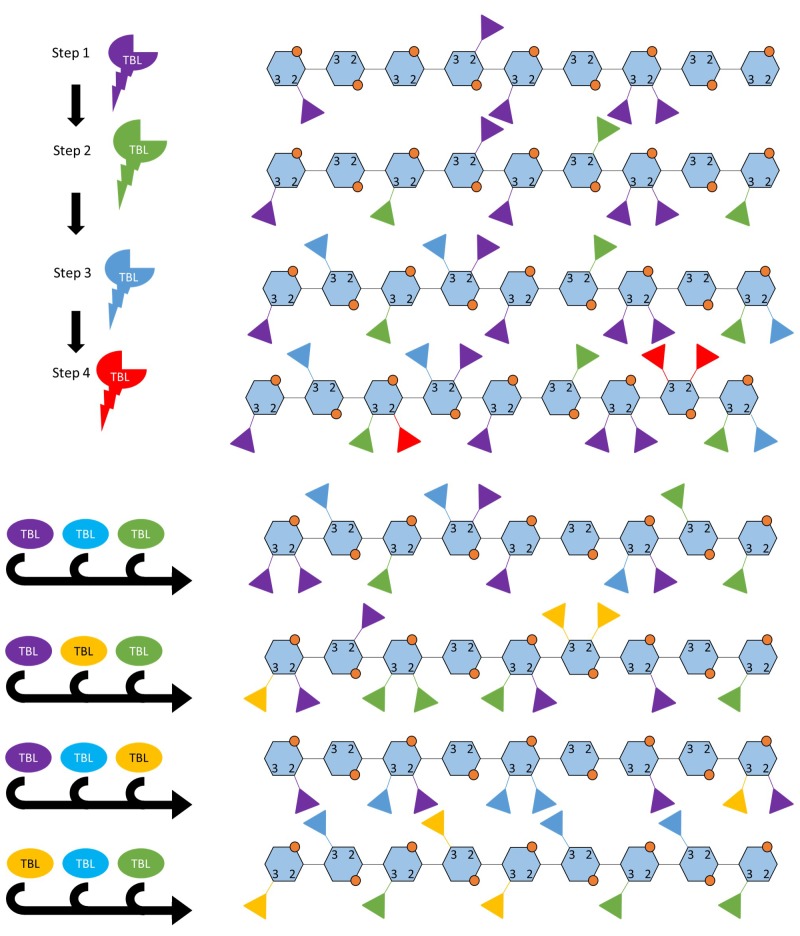
Two possible ways in which TBLs may establish the acetylation pattern. A highly simplified hypothesis of how acetylation patterns may be established by TBL proteins, this model does not incorporate interactions with glucuronic acids or post-synthesis modification by acetyl esterases. **(A)** Acetylation pattern may occur in a hierarchical or sequential manner where TBL genes are expressed in a certain order for the final pattern to be obtained or the enzyme kinetics determines the order in which the TBL proteins modify the backbone. **(B)** Certain patterns of acetylation occur when a combination of TBL proteins are present, these TBLs either interact with each other or share transcriptional regulation. The acetylation patterns shown here are hypothetical in order to illustrate the concept. Each TBL of a different color refers to a different TBL.

Xylan acetylation is a key factor in recalcitrance. Earlier biotechnology approaches aimed to reduce acetyl content by either targeting the genes involved in acetylation, or by removing the acetyl using ectopically expressed esterases (Manabe et al., 2013; Pogorelko et al., 2013; Xiong et al., 2013). Lately interest has shifted to understanding the effect of acetyl content on SCW traits as well as on plant physiology. The current hypothesized mechanism of xylan acetylation entails cytosolic acetyl-CoA being transported into the Golgi by a REDUCED WALL ACETYLATION (RWA) transporter, where it is sequestered by ALTERED XYLOGLUCAN 9 (AXY9) for polysaccharide acetylation, and subsequently used by xylan acetyltransferases in the TRICHOME BIREFRINGENCE-LIKE (TBL) family to acetylate the xylan backbone (Manabe et al., 2013; Xiong et al., 2013; Yuan et al., 2013; Schultink et al., 2015; Figure 1, 3). Moderate reductions in xylan acetylation (13–20%) through RWA downregulation or the introduction of an acetyl xylan esterase (AnAXE1) from *Aspergillus niger* has proven to reduce recalcitrance and alter SCW traits in an industrially beneficial way without impacting plant growth (Pawar et al., 2017a,b). Interestingly, plants with increased acetyl content also display reduced recalcitrance in addition to increased growth and stem volume (Yang et al., 2017). Therefore, increasing xylan acetyl content may be an approach to alter SCW traits, increase growth and reduce recalcitrance factors such as LCCs (Giummarella and Lawoko, 2016; Martínez-Abad et al., 2018). Biorefinery applications centered on acetic acid production from biomass may also benefit from biomass with higher acetyl content (Patil et al., 2013; Audu et al., 2018; Figure 1). Furthermore, acidic dissolving pulp production (DPP) benefits from the additional pH decrease caused by released acetyl groups (Chen et al., 2010; Xiao et al., 2015). It has been found that excess acetylation is processed by an esterase in monocots and would be valuable to determine whether this function is shared with dicots (Scheller, 2017; Zhang et al., 2017). Together these findings suggest that total xylan acetyl content is an important target for DPP and biorefinery applications in particular, but xylan acetylation patterns may be equally important and may explain some of the apparently discordant findings from up and down-regulation of xylan acetylation genes.

The nine functionally validated acetyltransferases associated with xylan acetylation all belong to a single clade in the TBL protein family. They all can add acetyl groups to O-2 and O-3 positions on the xylose residue, but differ in enzyme kinetic rate and display differing “preference” for the type of acetylation pattern that is produced suggesting that they differ in regiospecificity (Xiong et al., 2013; Yuan et al., 2013, 2016a,b,c). How the acetylation pattern is established is not fully understood and may be a complex process involving acetyl-CoA precursor supply, differential expression and activity of various xylan biosynthetic proteins and, potentially, editing upon delivery to the SCW. However, based on functional studies of TBL proteins, the activity of these proteins seems to have the largest effect of xylan acetylation (Xiong et al., 2013; Yuan et al., 2013, 2016a,b,c). How these proteins could establish a basal acetylation pattern could possibly include either (i) a hierarchical organization where the TBLs modify the xylan backbone in a specific order, or (ii) a combinatorial mechanism where different combinations of TBLs yield different acetylation patterns, or (iii) a combination of these that is accomplished by fine temporal and spatial transcriptional regulation (Figure 3). Some support for these hypotheses exist as only *esk1* (*TBL29*) mutations result in a large reduction in xylan acetylation and an *irx* phenotype, whereas other TBL mutants primarily exhibit changes in modification patterns. Additionally, TBL32 and TBL33 require a pre-existing α1,2-(Me)GlcA modification to acetylate the backbone (Xiong et al., 2013; Yuan et al., 2016a,b,c) hinting at an ordered process. Crystal structures of modification enzymes related to xyloglucan and alginate have provided valuable insight into possible mechanisms employed to obtain modification patterns (Sychantha et al., 2017; Culbertson et al., 2018). Acetylation patterns can potentially be established when the xylan enters a channel containing multiple TBLs. Alternatively, the TBL with the highest kinetic rate may preferentially perform the first modification and thereby initiate the pattern. As the xylose chain subsequently increases in length, other TBLs may alternatingly add acetyl groups to extend the pattern. Differentially regulating the TBL genes could yield different combinations of TBL proteins producing different modification patterns, a possibility that could be leveraged in biotechnology approaches to achieve desired acetylation patterns and biomass processing traits.

Transcriptional regulation of xylan acetylation related genes affects total acetyl content, the tissue in which acetylation occurs and the developmental stage at which acetylation occurs. Expression profiles of RWAs seem to determine acetyl-CoA supply for polysaccharide acetylation, as constitutively expressed RWAs from the AB clade supply acetyl-CoA to xyloglucan whereas the SCW expressed RWAs of the CD clade supply acetyl-CoA to xylan (Pawar et al., 2017b). Downregulation of RWA genes was shown to have no effect on plant growth while reducing recalcitrance and altering SCW composition (Pawar et al., 2017b). Overexpression of RWA was specified as having increased sugar release and yield (Macaya-Sanz et al., 2017). It is not currently known whether all TBLs which facilitate SCW xylan acetylation have been identified, but it is known that other TBL clades acetylate different polysaccharides such as xyloglucan (Vogel et al., 2004; Bischoff et al., 2010; Gille et al., 2011b). In the case of xyloglucan, two proteins (TBL22 and TBL27) are involved that perform the same function, but are expressed in different tissues (Gille et al., 2011b). The *Populus* homolog of TBL45 may be an example of this phenomenon, as it seems to add acetyl to xylan but is highly expressed in phloem and young leaf as opposed to xylem, thereby potentially adding an additional layer of complexity to how acetylation patterns emerge (Yang et al., 2017). In theory, an even large variety of acetyl patterns could be achieved by altering the expression of various combinations of TBLs and overexpressing RWA to provide sufficient acetyl-CoA precursor.

#### Methylglucuronic Acid Modifications

α1,2-linked D-GlcA modifications that have also been methylated at the O-4 position (MeGlcA) are the only xylan modification currently known to discriminate between the major and minor domains of xylan, PCW and SCW, as well as being associated with recalcitrance and SCW traits. The xylan backbone is modified with GlcA groups exclusively at the O-2 positions by GUX proteins which use UDP-GlcA as precursor, where GUX1 modifies the backbone in an even manner (major domain), GUX2 modifies in a closely and unevenly spaced manner (minor domain) while GUX3 modifies PCW xylan (Mortimer et al., 2010, 2015; Lee et al., 2012a; Bromley et al., 2013). Methylation of GlcA is carried out by GXM proteins from the DUF579 family by using the SAM precursor (Lee et al., 2012b; Urbanowicz et al., 2012; Song et al., 2016; Figure 1), with this type of methylation being exclusive to SCW xylan (Mortimer et al., 2015; Ishii et al., 2017). The ratio of methylated GlcA to unmethylated GlcA is dependent on the kinetic rate of GXM and differs between species (Yuan et al., 2014). What is less clear is the timing of GXM expression. It was found that 35S overexpression of GXM did not have an effect, but lignin linkages and LCCs were not investigated. The biological function of GlcA methylation is currently unclear, but it is known that its presence results in the xylan being more hydrophobic and has been suggested to be related to lignin composition and non-covalent interactions between xylan and lignin (Busse-Wicher et al., 2016b). However, reductions in MeGlcA groups lead to increased xylose release and a higher proportion of S-lignin (Urbanowicz et al., 2012). These groups could be taken advantage of for altering xylan’s association with cellulose through its two domains as well as its influence on lignin composition. This could potentially be achieved by using different SCW promoters rather than general overexpression with 35S to have a more specific effect during wood formation.

Some unknowns still exist in terms of these modifications namely the transporters needed for SAM and GlcA import into the Golgi during xylogenesis as well as the function of a DUF579 member IRX15. A uronic acid transporter which functions in seed mucilage has recently been identified (Saez-Aguayo et al., 2017). SAM transport into the Golgi has been detected and SAM transporters to the mitochondrion have been found (Palmieri et al., 2006; Ibar and Orellana, 2007). IRX15 and IRX15L are members of the DUF579 protein family, but their functions are not currently known, however, mutagenesis of a single gene leads to improved biomass processing (Brown et al., 2011; Jensen et al., 2011). Mutagenesis of both genes leads to an *irx* phenotype, shorter xylan chains, a corrugated SCW and an S3 layer which detaches from the S2 layer (Brown et al., 2011; Jensen et al., 2011). It is not currently known whether any editing of the (Me)GlcA group occurs in a manner similar to acetylation in monocots, but it is possible that this may relate to the function of IRX15/IRX15L, or have to do with the transport of xylan from the Golgi to the cell wall (Brown et al., 2011; Jensen et al., 2011). Identification of transporters could allow us to alter precursor supply for GlcA and its methylation through regulating the expression of these genes, while understanding the function of IRX15 may allow us to exploit its function in strategies that target specific SCW layers.

#### Interaction Between Modifications

Several studies have shown that there is competition and interaction between the xylan machinery predominantly for the modification of the O-2 position of xylose residues. Mutations in several genes related to xylan acetylation result in increased (Me)GlcA content (*tbl32 tbl33* is the exception) and *vice versa* for *gux* mutants (Busse-Wicher et al., 2014; Lee et al., 2014). The fact that most TBLs can modify the O-2 position is the reason why GlcA increases when these TBLs are mutated, albeit, not as drastically as in the *esk1* (*tbl29*) mutant (Xiong et al., 2013; Yuan et al., 2013, 2016b,c). The interaction between GlcA addition and acetylation is evident from the existence of xylose residues with 3-O acetylation and α1,2-(Me)GlcA. This pattern is produced by the action of TBL32 and TBL33 that add 3-O acetyl groups only to residues where the O-2 position was already occupied by(Me)GlcA (Yuan et al., 2016a; Zhong et al., 2017). The prevalence of these 3-O acetyl modifications increases in several *tbl* mutants in proportion to the increase of (Me)GlcA at O-2 positions (Yuan et al., 2013, 2016b,c). This residue is found to be the most recalcitrant to esterase treatment and it was found that a separate esterase from *Flavobacterium johnsoniae* is required to cleave this 3-O acetyl (Razeq et al., 2018; Puchart et al., 2019), perhaps suggesting a role in defense against certain types of pests which do not possess the specific esterase. These results become increasingly important when considering the processability of *Populus* and *Eucalyptus* wood as they both contain high proportions of this recalcitrant modification (Evtuguin et al., 2003; Puchart et al., 2019). The function of the 3-O acetylation/α1,2-(Me)GlcA modification pattern is unknown, but it may be linked to defense, or, alternatively, its proximity to (Me)GlcA might have a role in xylan’s association with lignin and it could therefore be a valuable biotechnology target.

An interesting interaction has been noted between GUX1 and ESK1 that may have broader implication for xylan modification. This relates to the types of modification patterns that could be achieved, as well as how xylan modification domains are formed. It has been suggested that GUX1 function relies on ESK1 in order to add GlcA modifications in an evenly spaced manner required for formation of the major domain that associates with the hydrophilic face of cellulose (Grantham et al., 2017). However, the *esk1* mutant can be complemented by expressing GUX1 under control of the ESK1 promoter (Xiong et al., 2015). This may indicate either that the timing of ESK1 expression is vital for the establishment of the even modification pattern of the major domain, or that the kinetic rate of GUX1 alone is insufficient to establish the major domain pattern in *esk1* (Rennie et al., 2012; Zhong et al., 2017). The increased expression under the control of the ESK1 promoter probably resolves this problem by increasing GUX1 protein abundance. Furthermore, it has been noted that O-2 modification, regardless of the type of modification, is vital for xylan function suggesting that these two modifications, at least in the context of xylan’s interaction with cellulose, fulfill equivalent functions (Xiong et al., 2015; Pereira et al., 2017) that could be exploited in biotechnology strategies to produce trees with different xylan properties and interactions.

It appears that ESK1 and GUX1 are required for major domain formation, but it still needs to be established whether other TBLs are essential for major domain formation, and whether any of the TBLs function specifically together with GUX2 to specify the minor domain (Bromley et al., 2013; Zhong et al., 2017). The *gux1 gux2* double mutant has xylan that is devoid of GlcA (i.e., only acetylated; Busse-Wicher et al., 2014; Lee et al., 2014). Conversely, knocking out all SCW xylan associated TBL genes, and expressing GUX genes under the appropriate promoters would lead to xylan with only (Me)GlcA modification. It would be interesting to contrast the SCW properties and composition of plants with xylans that exclusively harbor one type of modification, and determine whether such modified xylans are favorable for specific lignocellulosic biomass applications (Giummarella and Lawoko, 2016; Escudero et al., 2017; Pawar et al., 2017a; Figure 1). The stunted growth of the *esk1* mutant complicates efforts to study its effect on SCW composition (Xiong et al., 2013; Yuan et al., 2013). These results indicate that *ESK1/TBL29* may be the principal TBL for xylan acetylation, whereas the other TBLs may be providing additional complexity and functionality to the acetylation pattern. If that is indeed the case, the *esk1 kaktus* (Bensussan et al., 2015) as well as *esk1 max 4-7* (Ramírez et al., 2018) double mutants, which exhibit wild type growth and restored vessel morphology, while retaining the altered SCW phenotype of *esk1*, could be used as genetic backgrounds in a pursuit to completely remove acetyl from the xylan backbone. The rest of the xylan associated TBLs could be knocked out and the *TBL* gene promoters could be used to drive additional GUX gene expression to yield a plant with only (Me)GlcA modifications. These plants may be highly beneficial for biorefinery applications focused on extracting GlcA for the production of value added products such as glucaric acid, other GlcA derivatives and breakdown products (Zhao et al., 2007, 2013; Gürbüz et al., 2012;Salamone et al., 2014; Li et al., 2018; Figure 1). The identification of more mutants that can rescue *irx* phenotypes has the potential to allow the study and engineering of SCW and processing traits in plants with highly altered SCW compositions.

### Metabolic Engineering of Xylan Traits

#### Modeling the Interaction Between Xylan Biosynthesis and Other Cellular Processes

Xylan biosynthesis and modification relies on metabolic precursors from primary metabolism including nucleotide sugars, SAM and acetyl-CoA. How metabolism is co-regulated with SCW cell wall formation has previously been investigated for lignin and cellulose biosynthesis (Vanholme et al., 2012; Mizrachi et al., 2013; Yen et al., 2013), but this has not been done for xylan biosynthesis. The valuable insight that systems biology approaches provide may be applied to xylan modification potentially providing insight into how regulation differs between major domain formation required for xylan’s association with cellulose, and minor domain formation required for xylan’s association with lignin (Busse-Wicher et al., 2014; Marriott et al., 2016; Simmons et al., 2016; Peralta et al., 2017). Modification genes associated with the spatial and temporal changes in xylan modification throughout SCW development may be identified, and different SCW traits may arise from re-wiring the expression of these genes. It is known that plant metabolism is extensively altered during SCW development reflecting the need to maintain metabolic homeostasis in developing xylem cells (Li Z. et al., 2016; Ohtani et al., 2016). Understanding how xylan modification is coordinated with precursor metabolic pathways in developing xylem cells may highlight which pathways are the main sources of metabolic precursors such as SAM or acetyl-CoA, and whether alternative metabolic sources are used during different stages of SCW development.

Secondary cell wall formation is a very strong carbon sink in xylem cells and it is imperative that metabolic pools are tightly regulated to ensure optimal resource allocation between normal cellular metabolism and SCW biopolymer synthesis (Mizrachi and Myburg, 2016). Systems biology and systems genetics analyses are network based approaches that involve incorporating multiple “omics” data sets such as genomic, transcriptomic, metabolomic and proteomics data in order to model a biological process of interest (Guerriero et al., 2013). Each of these datasets represent variation in the components of the system, and using network based approaches, associations within and between systems components can be identified, providing a more holistic understanding of regulatory interactions and metabolic interdependencies (Kitano, 2002; Jansen, 2003; Kohl and Noble, 2009). Looking at these metabolic and regulatory processes, and their interactions, by comparing multiple systems components allows identification of emergent properties of the system that could not be otherwise observed (Feltus, 2014; Hillmer, 2015). Systems biology approaches rely on multi-omics experiments, typically on the same genotype that is subjected to different conditions (Barah et al., 2013; Rasmussen et al., 2013), sampled during different developmental stages (Li Z. et al., 2016), and/or creating sequential knock-out lines of many genes involved in a process of interest and observing how the system changes in response to each perturbation (Vanholme et al., 2012). This aids in understanding the dynamics of the process and which components are key targets for perturbation (Chen et al., 2014; Wang et al., 2014; Taylor-Teeples et al., 2015).

Systems genetics relies on using hundreds of genotypes in structured populations, where genetic variation acts as the main perturbation on the system (Civelek and Lusis, 2014). Besides systems components such as transcriptomics, metabolomics or proteomics, systems genetics can also link these components to complex trait variation in a population, for example SCW content and composition, industrial processing efficiency, physiological development and ecological adaptations, all in the context of genetic variation (Drost et al., 2010; Mizrachi et al., 2011, 2017; Civelek and Lusis, 2014; Christie et al., 2017). Individuals used for systems genetics analysis are typically phenotypically normal, therefore, the analysis provides valuable insight into the limits of natural perturbation that the system can handle, whereas mutagenesis of genes in systems biology approaches may be too extreme, leading to associations not found to occur normally. Genomic loci (represented by expression QTLs) associated with multiple components and/or traits, usually represent regulation hotspots with variants usually being found in hub genes or genes responsible for the biosynthesis of widely used metabolic precursors (Porth et al., 2013; Civelek and Lusis, 2014; Christie et al., 2017; Mizrachi et al., 2017). Networks generated from systems genetics analysis can be used to understand directionality of the associations in the system, and be used to understand epistatic interactions and pleotropic effects, which is highly beneficial for understanding highly coordinated processes such as SCW formation (Mizrachi and Myburg, 2016). Both approaches are valuable for identifying novel genes associated with a process, understanding interactions among systems components and for predicting the outcomes of genetic- and metabolic engineering strategies (Thumma et al., 2005; Mizrachi et al., 2011; Mottiar et al., 2016). A common finding in systems level analyses is that primary metabolic pathways are tightly coordinated with the various biosynthetic processes for which they provide precursors and that variation within the abundance of these pathways is associated with variation in complex traits (Vanholme et al., 2012; Li Z. et al., 2016; Ohtani et al., 2016; Mizrachi et al., 2017). Systems biology and genetics approaches may therefore be useful to understand how variation in precursor metabolic pathways affect xylan biosynthesis, the modification patterns, and how this variation ultimately impacts SCW and industrial processing traits.

#### Nucleotide Sugars

**FIGURE 4:**
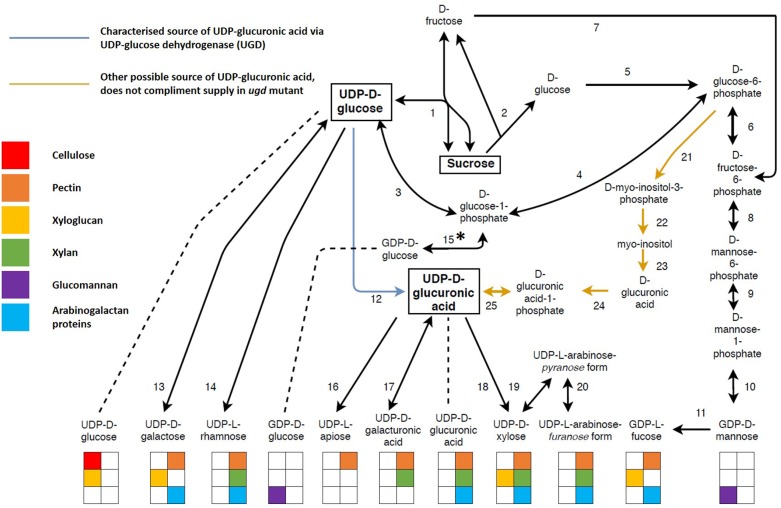
The interconversion of sugar nucleotides derived from sucrose and their use in biopolymer biosynthesis. Sugar nucleotides are the precursors required for the biosynthesis of both primary and secondary cell wall biopolymers with most sugar nucleotides being used for more than one biopolymer. Sucrose is the main source of nucleotide sugars. Its reversible cleavage by sucrose synthase yield D-fructose and UDP-glucose. UDP-glucose serves as a precursor for many other nucleotide sugars, either as a direct source (UDP-galactose, UDP-rhamnose and UDP-glucuronic acid) or an indirect source via from UDP-glucuronic acid (UDP-xylose, UDP-galacturonic acid and UDP-apiose). UDP-glucuronic acid can either be derived from UDP-glucose (blue arrow) or glucose-6-phospate (orange arrows). The former pathway (12) is an almost exclusive source of UDP-glucuronic acid, whereas the latter pathway (21–25) is understudied and is unable to complement an *ugd* mutant. ^∗^The enzyme required for this reaction has not been identified *in planta*. The coloring of the blocks under each nucleotide sugar indicates the biopolymer for which the nucleotide sugar is a precursor. Unidirectional arrows indicate irreversible reactions whereas bidirectional arrows indicate reversible reactions. Key metabolites are highlighted in square boxes. The enzymes (1–25) represented by each number can be found in Supplementary Table S1, along with additional information such as gene ID and cellular localisation.

The interconversion of nucleotide sugars is vital for xylan biosynthesis. All nucleotide sugars required for xylan biosynthesis are derived from UDP-Glc (Sharples and Fry, 2007; Bar-Peled and O’Neill, 2011; Reboul et al., 2011), produced together with D-fructose through the reversible action of SUCROSE SYNTHASE (SuSy) (Amor et al., 1995; Salnikov et al., 2001; Coleman et al., 2009; Figure 4 and Supplementary Table S1). In order to effectively partition these sugars between these polysaccharides, some form of co-regulation needs to exist, and it is already known that xylan and cellulose biosynthesis are transcriptionally co-regulated in order to balance UDP-Glc usage for cellulose and xylan via UDP-Xyl (Brown et al., 2005; Ko et al., 2006). Xylan and glucomannan are the two main hemiceluloses present in the SCW (Plomion et al., 2001), but they do not directly compete for substrate. GDP-mannose required for glucomannan biosynthesis is D-fructose derived, while the irreversible sucrose breakdown by INVERTASE (2) produces D-glucose required for the biosynthesis of GDP-glucose, however, this pathway still needs to be characterized *in planta* (Hinman and Villemez, 1975; Sharples and Fry, 2007; Bar-Peled and O’Neill, 2011). Two strategies for carbon allocation during SCW formation exist, transcriptional co-regulation when the metabolic precursor is shared (e.g., cellulose and xylan), or using precursors that are not derived from competing metabolic pathways (e.g., xylan and glucomannan).

Apart from xylan biosynthesis, sugar nucleotides are used for the biosynthesis of PCW polysaccharides xyloglucan and the pectins, HG, RGI and RGII (Mølhøj et al., 2003; Mohnen, 2008; Bar-Peled and O’Neill, 2011; Atmodjo et al., 2013; Saez-Aguayo et al., 2017). UDP-sugars may be allocated to xyloglucan, pectin and xylan through spatial and/or temporal regulation, as during the transition from primary to secondary growth in developing xylem, downregulation of genes associated with PCW polysaccharides is observed (Hertzberg et al., 2001). Additionally, transporters can serve as key points of regulation through differential transport activity (Ebert et al., 2015), process specificity (Manabe et al., 2013; Mortimer et al., 2013), alternate functions in different tissues (Saez-Aguayo et al., 2017), or through interactions with the biosynthetic machinery (Gille and Pauly, 2012). In the case of xyloglucan, the cellulose synthase-like C4 (CSLC4) enzyme acts as the biosynthetic machinery and the transporter. Catalysis of the glucan chain begins in the cytosol and the chain is elongated directly into the Golgi lumen by passing through the membrane spanning CSLC4 protein (Davis et al., 2010; Chou et al., 2012). Spatial regulation may be a manner in which to avoid competition for precursors between the synthesis of PCW and SCW polysaccharides, whereas the differential activity of transporters and biosynthetic proteins may be a mechanism by which adequate precursor supply is ensured for Golgi localized polysaccharide synthesis. These mechanisms would be important avenues for metabolic engineering approaches targeted at nucleotide sugar interconversions. More than one polysaccharide could be affected, however, unwanted polysaccharide accumulation or deleterious effects could be avoided by making use of some of the aforementioned regulatory mechanisms.

#### S-Adenosylmethionine

**FIGURE 5:**
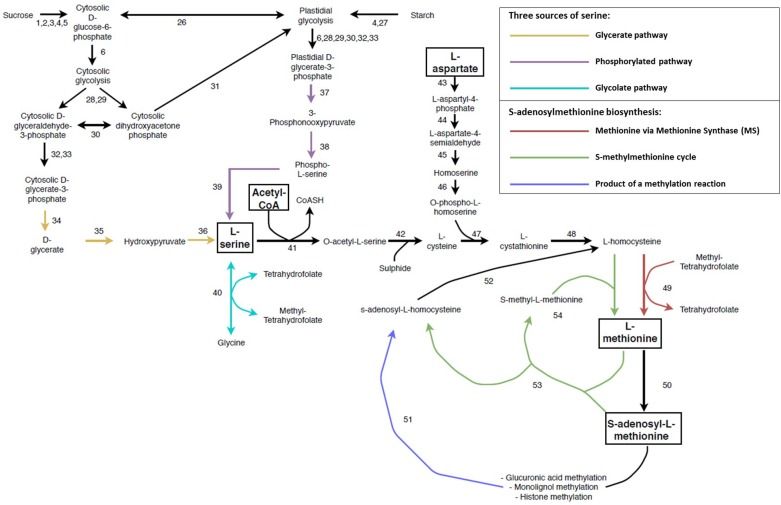
Biosynthesis and cycling of s-adenosylmethionine. For s-adenosylmethionine (SAM) to be produced, methionine needs to be biosynthesised first which requires sulfide, the cofactor acetyl-CoA as well as the two amino acids aspartate and serine. There are three possible sources from which serine can be derived, with the glycolate pathway (light blue) being predominant in autotrophic tissues and the phosphorylated pathway (purple) predominating in heterotrophic tissues, whereas the metabolic context of the glycerate pathway (orange) is still unknown. SAM SYNTHETASE (50) is the enzyme responsible for converting methionine into SAM (Peleman et al., 1989). SAM can then be used by countless methyltransferases, which in turn add a methyl to their target molecule, releasing S-adenosylhomocysteine (SAH) as a by-product (Roje, 2006; dark blue). The SAH released from these reactions is recycled through the action of SAH HYDROLASE (SAHH; 52; Rocha et al., 2005; Pereira et al., 2007) which yields adenosine and homocysteine that is subsequently converted to methionine by METHIONINE SYNTHASE (MS; 49; red) using a folate cofactor (Ravanel et al., 2004; Loizeau et al., 2007). Due to the importance of SAM, homeostasis needs to be maintained, which occurs through the action of SAM:METHIONINE S-METHYLTRANSFERASE (53) which converts SAM and methionine into S-methylmethionine (SMM) and SAH (green). SMM produced in the leaves, can be moved into the phloem and transported to different tissues. The accumulation of SMM and methionine in the seeds is done in both a spatial and temporal manner (Frank et al., 2015), but whether the SMM cycle is used in other non-reproductive tissues such as xylem during SCW formation is still unclear. This pathway is the most common source of SAM in seeds. SMM produced in the leaves arrives at the seeds via the phloem, and HOMOCYSTEINE S-METHYLTRANSFERASE (54) subsequently uses the SMM and homocysteine to produce two methionine molecules (Cohen et al., 2017). The accumulation of SMM and methionine in the seeds is done in both a spatial and temporal manner (Frank et al., 2015), but whether the SMM cycle is used in other non-reproductive tissues such as xylem during SCW formation is still unclear. Unidirectional arrows indicate irreversible reactions, bidirectional arrows indicate reversible reactions, whereas lines with multiple numbers next to it indicates multiple enzymatic reactions. Key metabolites are highlighted in square boxes. The enzymes (26–54) represented by each number can be found in Supplementary Table S1, along with additional information such as gene ID and cellular localisation. Several enzymatic steps are repeated from Figure 4.

SAM is the universal methyl donor in all forms of life. In SCW forming tissue, it is vital for the biosynthesis of ethylene and polyamines and for the methylation of GlcA modifications on the xylan backbones, monolignols as well as histones and DNA (Inoue et al., 1998; Shen et al., 2002; Roje, 2006; Do et al., 2007; Lee et al., 2012b; Urbanowicz et al., 2012; Sauter et al., 2013; Wang et al., 2016; Hussey et al., 2017). SAM is derived from methionine, which is produced through several conversion steps from the amino acids aspartate and serine, as well as the incorporation of acetyl-CoA (Jander and Joshi, 2009; Sauter et al., 2013; Ros et al., 2014; Figure 5 and Supplementary Table S1). The reliance on multiple key metabolites for its biosynthesis imposes a requirement for tight regulation of SAM biosynthesis. As opposed to bacteria, fungi and animals, there are three sources of serine biosynthesis in plants namely the glycolate pathway, cytosolic glycerate pathway and the plastidial phosphorylated pathway (Ros et al., 2014; Figure 5 and Supplementary Table S1). The glycolate pathway has been suggested as the most important pathway of serine biosynthesis and has been shown to have biotechnology applications. Transgenic *Populus* overexpressing SERINE HYDROXYMETHYLTRANSFERASE (40) displayed increased growth, stem diameter and sugar release as well as a decrease in lignin content (Zhang et al., 2019). The glycolate pathway is involved in the cycling of folate derivatives (Douce et al., 2001; Loizeau et al., 2007), but is limited to periods of sunlight (Ros et al., 2014). The phosphorylated pathway is the predominant pathway associated with non-green tissues (such as xylem and root; Cascales-Miñana et al., 2013; Toujani et al., 2013) and is also involved in nitrogen metabolism (Anoman et al., 2015). The L-serine from the glycolate pathway may move from the leaves through the phloem to non-photosynthetic tissues (Riens et al., 1991; Lam et al., 1995), thereby supplementing the L-serine derived from the phosphorylated pathway. Although the non-photosynthetic glycerate pathway has been shown to be functional (Kleczkowski and Givan, 1988; Greenler et al., 1989) it has been largely understudied. The 3-PHOSPHOGLYCERATE PHOSPHATASE (34) enzyme has only been partially purified (Randall et al., 1971) and the gene(s) responsible for this function are still to be identified in *Arabidopsis* (Cascales-Miñana et al., 2013).

SAM is used as a cofactor for countless methylation reactions. S-adenosylhomocysteine (SAH; Rocha et al., 2005; Pereira et al., 2007) is produced as a by-product but is reconstituted to SAM through the action of SAH hydrolase (52), methionine synthase (Ravanel et al., 2004; Loizeau et al., 2007) and SAM synthetase (SAMS; 50; Peleman et al., 1989). Previous studies have noted that 80% of methionine produced by the plant is used for SAM biosynthesis (Giovanelli et al., 1985) and the role of SAM is especially highlighted during germination, where methionine was observed to accumulate before being converted to SAM the following day (Gallardo et al., 2002). A similar process may occur during xylogenesis (Ohtani et al., 2016). SAM homeostasis as well as transport to reproductive tissues is through the action of the s-methylmethionine (SMM) cycle. SMM produced in the leaves, can be moved into the phloem and transported to different tissues to act as a source of SAM (Cohen et al., 2017). The accumulation of SMM and methionine in the seeds occurs in both a spatial and temporal manner (Frank et al., 2015), but whether the SMM cycle is used in other non-reproductive tissues such as xylem during SCW formation is still unclear. Homeostasis has been noted between monolignol methylation and xylan GlcA methylation as *gxm* mutants exhibit an increase in monolignol methylation, whereas reductions in both forms of methylation were observed when genes of the folate biosynthesis pathway were mutated (Urbanowicz et al., 2012; Srivastava et al., 2015). Homeostasis of SAM may be determined by the action of a yet undiscovered Golgi localized SAM transporter, which may be a reason why overexpression of GXM2 and GXM3 did not display an altered lignin composition opposite to that of the *gxm3* mutant (Urbanowicz et al., 2012; Yuan et al., 2014). Additionally, polysaccharide biosynthesis and methylation may influence SAM transporter activity. Golgi microsomes have been used to study SAM intake into the Golgi. Intake increased when a combination of either UDP-GlcA and acetyl-CoA or UDP-GalA and acetyl-CoA was present whereas intake of SAM decreased when SAH was present (Ibar and Orellana, 2007).

#### Acetyl-CoA

**FIGURE 6:**
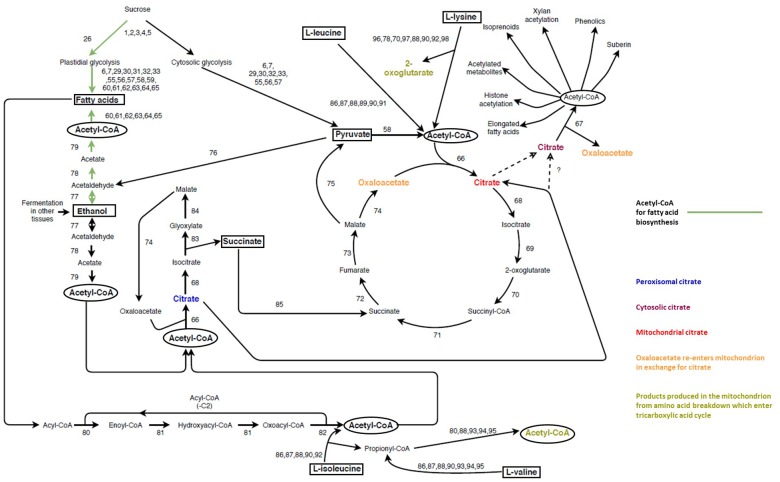
Multiple metabolic sources of plastidial and cytosolic acetyl-CoA. Plastidial glycolysis contributes pyruvate which is converted to acetyl-CoA by the plastidial Pyruvate Dehydrogenase (PDH) (58) complex for fatty acid biosynthesis (Johnston et al., 1997; Lin et al., 2003; green arrows) whereas cytosolic glycolysis is the main metabolic route which contributes pyruvate toward the TCA cycle for respiration in the mitochondrion (Luethy et al., 1995). In the mitochondrion, the pyruvate is broken down to acetyl-CoA which forms part of the TCA cycle through the action of CITRATE SYNTHASE (66) which produces mitochondrial citrate (red) from acetyl-CoA and oxaloacetate. Mitochondrial citrate (red) is transported to the cytosol (purple) and converted by heteromeric ATP-CITRATE LYASE (67) into acetyl-CoA and oxaloacetate. This is the main source of cytosolic acetyl-CoA pool whereas the resulting oxaloacetate is transported back into the mitochondrion in exchange for more citrate (orange). Acetaldehyde derived either from fermentation or from ethanol from other tissues can either enter the plastid where it contributes to fatty acid biosynthesis (green arrows), or be converted to acetate in the cytosol and enter the peroxisome. Acetyl-CoA can be generated in the peroxisome from acetate (79), β-oxidation of fatty acids (80–82), or breakdown of isoleucine (82, 86, 87, 88, 90, 92). The peroxisomal acetyl-CoA produced from the three aforementioned pathways can contribute to the cytosolic acetyl-CoA through the TCA either as citrate (blue) or succinate from the glyoxylate cyle (85). The breakdown of amino acids in the mitochondrion supplies various TCA cycle intermediates (gold) which can either contribute to respiration or the cytosolic acetyl-CoA pool. Unidirectional arrows indicate irreversible reactions, bidirectional arrows indicate reversible reactions whereas lines with multiple numbers next to it indicates multiple enzymatic reactions. Key metabolites are highlighted in square boxes, whereas acetyl-CoA is highlighted by ovals. The enzyme represented by each number can be found in Supplementary Table S1, along with additional information such as gene ID and cellular localization with steps 55–98 being displayed above. Several enzymatic steps are repeated from and Figure 4, 5.

Acetyl-CoA is a vital metabolite which acts as a universal acetyl donor for multiple processes such as fatty acid biosynthesis and the acetylation of molecules such as polysaccharides (xylan, xyloglucan, pectins and glucomannan), metabolites (phenolics and isoprenoids) as well as proteins (e.g., histones); (Pauly and Scheller, 2000; Fatland et al., 2005; Oliver et al., 2009; Chen C. et al., 2017). Compartmentalisation of acetyl-CoA between the plastid (fatty acids) and cytosol (latter mentioned processes) is a mechanism that is used to ensure that different competing processes receive adequate amounts of cofactor (Schwender et al., 2006; Faria-Blanc et al., 2018). The cytosolic ATP-CITRATE LYASE (ACL) (67) enzyme that converts citrate into oxaloacetate and acetyl-CoA is vital for cellular function as *acl* mutants are embryo lethal, whereas downregulation yields plants with “bonsai” phenotypes (Fatland et al., 2002, 2005; Figure 6 and Supplementary Table S1). The citrate required for this reaction is derived primarily from the mitochondrial tricarboxylic acid (TCA) cycle that obtains carbon skeletons via pyruvate from cytosolic glycolysis (Picault et al., 2002; Giegé et al., 2003). Plants have two glycolytic pathways (cytosolic and plastidial), each with its own distinct enzymes, and the pyruvate produced from glycolysis is essential for acetyl-CoA generation (Plaxton, 1996; Givan, 1999; Giegé et al., 2003; Schwender et al., 2006). Cytosolic glycolysis derives pyruvate from sugars such as UDP-Glc, glucose-6-phosphate (G-6-P) or fructose-6-phosphate (F-6-P) from sucrose breakdown (Plaxton, 1996; Givan, 1999; Giegé et al., 2003; Stein et al., 2016). Plastidial glycolysis uses cytosolic G-6-P, triose phosphates and phosphoenolpyruvate which are transported into the plastid by various transporters (Fischer and Weber, 2002; Schwender et al., 2003, 2006; Flores-Tornero et al., 2017). Alternatively starch breakdown can supply glucose-1-phosphate (Plaxton, 1996; Givan, 1999; Ho et al., 2017; Pick and Avidan, 2017). Although mitochondrial and plastidial glycolysis are the predominant sources of acetyl-CoA, other pathways have been found to contribute to both acetyl-CoA pools.

Under hypoxic and/or energy deficient conditions, fermentation pathways become active in order to keep glycolysis functioning by maintaining NAD+ generation which is accomplished by converting pyruvate to ethanol (Tadege et al., 1999; Zabalza et al., 2009). Two enzymes are involved in this conversion namely: PYRUVATE DECARBOXYLASE (76) which converts pyruvate to a toxic intermediate, acetaldehyde, and subsequently ALCOHOL DEHYDROGENASE (77) converts the acetaldehyde to ethanol, effectively regenerating NAD+ from NADH (Bucher et al., 1995; Tadege and Kuhlemeier, 1997; Tadege et al., 1999; Figure 6 and Supplementary Table S1). The ethanol is hypothesized to move through the vasculature from roots (hypoxic conditions) to the upper organs to be converted back into acetaldehyde and then into acetate by ALDEHYDE DEHYDROGENASE (78); (Kreuzwieser et al., 1999; Oliver et al., 2009). This acetate can enter fatty acid biosynthesis in the plastid (Mellema et al., 2002), but has been shown to not greatly contribute to this process (Lin and Oliver, 2008), or can enter the peroxisome and potentially contribute to the cytosolic acetyl-CoA pool through TCA (Turner et al., 2005; Allen et al., 2011; Figure 6). Acetyl-CoA produced in the peroxisome can exit in two ways, (i) the citrate enters the glyoxylate pathway by which succinate is produced and is able to rejoin the TCA or (ii) the citrate enters the TCA directly (Mettler and Beevers, 1980; Salon et al., 1988; Eastmond et al., 2000). It is, however, unknown whether citrate exiting the peroxisome can contribute directly to the cytosolic acetyl-CoA pool as a substrate for ACL. Besides acetate, β-oxidation and the degradation of isoleucine use this pathway in order to provide carbon skeletons to the TCA cycle (Baker et al., 2006; Hildebrandt et al., 2015; Figure 6 and Supplementary Table S1).

The breakdown of amino acids (especially BCAA and lysine) is an understudied source of acetyl-CoA in plants. The enzymes responsible still need to be identified as several of the degradation steps are currently inferred by homology to yeast, bacteria and mammals (Anderson et al., 1998; Schuster and Binder, 2005; Araújo et al., 2010; Hildebrandt et al., 2015; Figure 6 and Supplementary Table S1). The acetyl-CoA produced by these pathways has been shown to be required by oligeneous diatoms for the biosynthesis of fatty acids and the related genes become upregulated during late SCW formation in inducible VND7 *Arabidopsis* (Ge et al., 2014; Li Z. et al., 2016). Due to the importance of acetyl-CoA, the existence of so many biosynthetic pathways may provide redundancy in order to maintain the cytosolic acetyl-CoA pool under various developmental stages and environmental conditions. Alternatively, these pathways may be required to maintain TCA cycle activity when the flux of carbon in glycolysis is redirected to other processes such as phenylalanine biosynthesis (Ohtani et al., 2016). Xylan acetylation and the production of SAM for monolignol and GlcA methylation both use acetyl-CoA, which suggests that competition for acetyl-CoA occurs and needs to be regulated in some manner. It is possible that cytosolic acetyl-CoA pools are maintained by multiple pathways in order to reduce competition for the cofactor among xylan acetylation and the methylation of GlcA and monolignols. If true, identifying underlying regulatory mechanisms responsible for reducing cofactor competition could provide novel avenues to alter SCW traits through metabolic engineering.

## Conclusion

Xylan biosynthesis is a process that affects SCW formation, the composition and interaction of SCW components, as well as the efficiency of the deconstruction of lignocellulosic biomass for the production of various bioproducts. How perturbing xylan traits and modification patterns may affect SCW composition and processing still needs further investigation. Additional unknowns include the genes which code for the Golgi localized GlcA and SAM transporters, the exact functions of IRX15/IRX15L, the full complement of XSC and RES synthesizing proteins, and whether there are any more xylan associated proteins. Work in monocots suggest that several other proteins are involved in xylan biosynthesis and interact with the biosynthetic machinery (Zeng et al., 2010; Jiang et al., 2016). It still remains to be seen whether the interacting partners are conserved in other plant lineages. The presence of a UAM in the XSC may provide adequate UDP-Ara*f*, which could explain why rice XAT fails to add sufficient amounts of arabinose modifications to xylan in *Arabidopsis* (Zeng et al., 2010; Xiong et al., 2015; Jiang et al., 2016). The fact that hormone binding proteins were found to interact with the XSC may indicate that hormones dictate SCW composition through xylan biosynthesis. Furthermore, the finding that xylan mutants are often more tolerant to stresses may be linked to altered interactions between the XSC and interacting proteins, which means that stress tolerant plants could be generated by manipulating xylan properties (Xin and Browse, 1998; Keppler and Showalter, 2010; Jiang et al., 2016; Escudero et al., 2017). In mutants such as *esk1* that exhibits altered metabolism, carbon flux may be redirected to upregulated defense pathways as opposed to SCW, affecting not only xylan but also other polysaccharides. This scenario may be occurring in other xylan mutants as well, which explains the reduction in the content of polysaccharides such as glucomannan and pectin.

The multiple proteins involved in xylan biosynthesis and modification, the requirement of different xylan conformations needed to associate with cellulose and lignin, as well as spatial and temporal changes in xylan modification during SCW development suggest that different combinations of enzymes may be needed during different SCW formation stages. Since xylan shares metabolic precursors with cellulose and lignin, co-regulation of not only structural genes, but also primary metabolism needs to occur for SCW formation to take place correctly. Systems biology and systems genetics approaches could identify which parts of the biosynthetic machinery are responsible for xylan-cellulose and xylan-lignin interactions, as well as the ultimate composition and structure of the two biopolymers. Such approaches will also aid in identifying the pathways that are vital for supplying precursors and that, when perturbed, might lead to changes in xylan modification patterns. The mechanisms behind the formation of the two xylan domains still needs to be understood, as well as what effect supply of metabolic precursors has on establishing these domains. Such understanding would inform metabolic engineering efforts aimed at altering SCW properties. The only currently known effect of metabolism on xylan biosynthesis is that a stronger flux of sucrose increases total xylan content, and that xylan biosynthetic rates are temporally linked to the sucrose produced in the leaves and supplied to the xylem via the phloem (Ishihara et al., 2015).

Metabolic engineering approaches have the potential to target multiple processes at once, using genetic modifications that can be applied in a tissue-specific manner, to yield the trait of interest without causing undesirable effects on plant growth and defense. This approach has been applied successfully to increase SCW biomass, increase lignin content, alter lignin composition and reduce methyl content in the SCW, all of which displayed beneficial properties for the process of interest (Huntley et al., 2003; Srivastava et al., 2015; Eloy et al., 2017; Fan et al., 2017; Zhang et al., 2019). CRISPR gene editing could prove to be a useful tool for metabolic engineering, as it has been successfully applied to alter SCW traits (Fan et al., 2015; Zhou et al., 2015; Park et al., 2017) and provides the option to either knock-out or alternatively regulate genes involved in interdependent metabolic processes (Jinek et al., 2012; Qi et al., 2013; Vojta et al., 2016; Xiong et al., 2017). Additionally, knocked-out or downregulated pathway steps could be replaced with alternative enzymes to “rewire” metabolism in ways that would achieve desirable industrial processing traits without disrupting plant growth and defenses (Aznar et al., 2018). Finally, greater understanding of xylan biosynthesis in diverse plant lineages could drive successful introduction of novel modifications to fundamentally alter SCW composition in woody biomass. Producing a tree with an ideal biomass composition for all industrial applications might not be possible. However, xylan bioengineering may be an approach whereby woody biomass with customized composition can be created for different biorefinery application.

## Author Contributions

MW conceived, wrote and made figures for the review. VM, EM, and AM contributed to the outline of the review, and provided extensive edits to both the text and figures.

## Conflict of Interest Statement

The authors declare that the research was conducted in the absence of any commercial or financial relationships that could be construed as a potential conflict of interest.

## Supplementary Material

TABLE S1 List of enzymes which catalyze the biosynthesis of sugar nucleotides, s-adenosyl methionine and acetyl-CoA. (a) The numbering of the enzymes corresponds to Figure 4–6. (b) Name of the enzyme. (c) Not all accessions were provided for each reaction, just one representative from each cellular compartment. Accessions and cellular localisation were obtained from TAIR and SUBA3, respectively.

FILE S2 References pertaining to numbered items in Table 1.

FILE S3 Biotechnology approaches that have scaled from *Arabidopsis* to *Populus*.
